# Adipose tissue and body composition in women six years after gestational diabetes: factors associated with development of type 2 diabetes

**DOI:** 10.1080/21623945.2018.1521230

**Published:** 2018-09-30

**Authors:** Henrik Svensson, Louise Wetterling, Ulrika Andersson-Hall, Eva Jennische, Staffan Edén, Agneta Holmäng, Malin Lönn

**Affiliations:** a Department of Clinical Chemistry and Transfusion Medicine, Institute of Biomedicine, Sahlgrenska Academy, University of Gothenburg, Gothenburg, Sweden; b Department of Physiology, Institute of Neuroscience and Physiology, Sahlgrenska Academy, University of Gothenburg, Gothenburg, Sweden; c Department of Medical Biochemistry and Cell Biology, Institute of Biomedicine, Sahlgrenska Academy, University of Gothenburg, Gothenburg, Sweden; d Department of Internal Medicine, Institute of Medicine, Sahlgrenska Academy, University of Gothenburg, Gothenburg, Sweden

**Keywords:** adipocyte fatty acid-binding protein, adipokines, adipose tissue, adipocyte size, BMI, body composition, gestational diabetes mellitus, prediabetes, type 2 diabetes mellitus, weight gain

## Abstract

Factors differentiating women at highest risk of progression to type 2 diabetes mellitus (T2DM) after gestational diabetes mellitus (GDM) are incompletely known. Our aim was to characterize adipose tissue and body composition in relation to glucose metabolism in women with a history of GDM and to identify factors associated with development of T2DM. We examined glucose tolerance (OGTT), insulin sensitivity (HOMA-IR), body composition (anthropometry, air displacement plethysmography), and blood chemistry in 39 women 6 years after GDM. An adipose tissue biopsy was obtained to assess the size, number, and lipolytic activity of adipocytes, and adipokine release and density of immune cells and blood vessels in adipose tissue. Normal glucose tolerance (NGT) was identified in 31 women and impaired glucose metabolism (IGM) in 8. Women with IGM had higher BMI/fat mass, and related expected adipose tissue features, than women with NGT. Ethnicity was similar in the groups, but numerically there was a higher proportion of European women in the NGT group and a higher proportion of non-European women in the IGM group. BMI was the best discriminator of NGT versus IGM (multivariable logistic regression: OR = 1.34, *P *< 0.01). Waist-to-height ratio and adipocyte volume were most strongly associated with HOMA-IR (multivariable linear regression: R^2^ = 0.656, *P *< 0.001). After adjustment for BMI/ethnicity, women with IGM had increased serum adipocyte fatty acid-binding protein, weight gain after index pregnancy, and a lower proportion of fat-free mass. These factors, together with high BMI, abdominal fat distribution, and enlarged adipocytes, may increase the risk of progression to T2DM after GDM.

## Background

The prevalence of gestational diabetes mellitus (GDM), defined as any degree of glucose intolerance with onset or first recognition during pregnancy,^1^ has risen in parallel with the worldwide increase in the prevalence of obesity,^2^ a well-known risk factor for GDM.^3,4^ GDM affects 1–18% of all pregnancies, partly depending on the diagnostic criteria used and the ethnicity of the population sampled.^5^

Women with a history of GDM have an increased risk for impaired glucose tolerance (IGT) (i.e., prediabetes) and type 2 diabetes mellitus (T2DM) later in life.^6,7^ The frequency of progression from GDM to T2DM varies among studies, in part reflecting differences in the diagnostic criteria for postpartum diabetes and in the length of follow-up. However, after GDM, the risk for T2DM in the future is seven-fold higher than after a normoglycemic pregnancy.^6^ Factors differentiating women at highest risk of progression to T2DM after GDM are incompletely known. Weight-related characteristics reported to be associated with progression to T2DM include high pre-pregnancy BMI, weight gain after GDM, and most recent BMI after GDM pregnancy.^8–10^ Other important factors are high fasting glucose during pregnancy, early GDM diagnosis, and ethnicity.^11–13^ Besides these risk factors, maternal age, a family history of diabetes, parity, HbA1c, and insulin treatment during pregnancy were listed in a recent meta-analysis as maternal characteristics, and factors specific to pregnancy, associated with future onset of T2DM.^14^

Although weight gain and a high BMI are strongly associated with risk of progression to IGT or T2DM after GDM, no study has addressed the role of adipose tissue characteristics in this context. In this study, we collected abdominal subcutaneous adipose tissue biopsies from women 6 years after diagnosis of GDM. Our aims were to characterize adipose tissue morphology/function and body composition in relation to glucose metabolism at follow-up and to identify factors associated with development of T2DM in women with a history of GDM.

## Results

Thirty-nine women with previous GDM were included and examined at the first and second follow-up study visits scheduled 5.8 ± 0.50 and 5.9 ± 0.52 years after GDM diagnosis. Based on fasting glucose and 2-h glucose in an oral glucose tolerance test (OGTT), normal glucose tolerance (NGT) was identified in 31 women, impaired fasting glucose (IFG) in one, IGT in three, IFG plus IGT in two, and previously undiagnosed T2DM in two. Thus, eight women had impaired glucose metabolism (IGM).

### Characteristics related to index pregnancy

Characteristics related to the index pregnancy with GDM diagnosis are presented in Table 1. Women with IGM at follow-up weighed more than women with NGT and had a higher BMI early in the index pregnancy. The two groups were similar in age, parity, previous GDM, ethnicity (although numerically there was a higher proportion of European women in the NGT group and a higher proportion on non-European women in the IGM group), heredity (first-degree relative), gestational weight gain, fasting and OGTT 2-h glucose at GDM diagnosis, proportion of women with early GDM diagnosis, and proportion of women treated with insulin.

**Table 1.** Characteristics related to index pregnancy with GDM diagnosis.

	GDM at index pregnancy (n = 39)		
Characteristic	NGT at follow-up (n = 31)	IGM at follow-up (n = 8)	*P* ^a^
Age, y	33.7 ± 4.4	33.9 ± 4.2	1.000
Parity, n	1.2 ± 0.8	1.1 ± 1.0	0.746
GDM pregnancy before index pregnancy, %^d^	29	33	1.000^c^
Ethnicity, % (Europe/non-Europe/unknown)	58/32/10	38/62/0	0.299^b^
Heredity, % (yes/no/unknown)^e^	23/71/6	25/75/0	1.000^b^
Weight early pregnancy, kg^f^	73.1 ± 13.9	84.5 ± 16.3	**0.048**
BMI early pregnancy, kg/m^2f^	26.8 ± 4.4	31.1 ± 5.5	**0.044**
Gestational weight gain, kg^g^	9.6 ± 4.1	9.9 ± 7.6	0.825
P-glucose, mmol/l^h^	4.8 ± 1.4	5.4 ± 0.6	0.059
P-glucose 2h OGTT, mmol/l^h^	10.9 ± 0.8	11.5 ± 1.3	0.300
Diagnosis < 140 days of gestation, %	19	25	0.658^c^
Insulin treatment, %	10	25	0.268^c^

Values are mean ± SD or percentage. ^a^ Independent samples Mann-Whitney U test. ^b^ χ^2^ test. ^c^ Fisher’s exact test. ^d^ Number of pregnancies before index pregnancy: 24 (NGT), 6 (IGM). ^e^ First-degree relative. ^f^ In gestational week 12.2 ± 2.9. ^g^ Determined between first and last maternity care visits. ^h^ At GDM diagnosis.

### Whole-body characteristics at follow-up

At follow-up 6 years after GDM diagnosis, fasting glucose was higher in the IGM group than in the NGT group (6.4 ± 0.4 vs 5.4 ± 0.4, *P *< 0.001), as was OGTT 2-h glucose (9.4 ± 2.4 vs 5.5 ± 1.0, *P *< 0.001). Other whole-body characteristics of the women are shown in Table 2. Women with IGM weighed more, had a higher BMI, weight gain after the index pregnancy, higher measures of abdominal fat distribution, a higher proportion of fat mass (FM), a lower proportion of fat-free mass (FFM), higher HOMA-IR, higher HbA1c, and higher serum levels of insulin, leptin, and adipocyte fatty acid–binding protein (AFABP) (Table 2). After adjustment for BMI, HbA1c and serum AFABP remained higher in women with IGM. After adjustment for both BMI and ethnicity, women with IGM weighed more than those with NGT, had gained weight after the index pregnancy, and had a higher HOMA-IR, higher serum levels of AFABP and high-sensitivity C-reactive protein (hsCRP), and a lower proportion of FFM (Table 2). The single factor consistently higher in the IGM group than in the NGT group was serum AFABP, both before and after adjustments for BMI and BMI/ethnicity.

**Table 2.** Whole-body characteristics of women at follow-up 6 years after GDM diagnosis.

	GDM at index pregnancy (n = 39)				
Characteristic	NGT at follow-up (n = 31)	IGM at follow-up (n = 8)	*P* ^a^	*P adj* ^b^	*P adj* ^c^
Age, y	39.5 ± 4.5	39.9 ± 4.3	0.878	-	-
Parity, n	2.5 ± 0.7	2.8 ± 1.3	0.720	-	-
GDM pregnancy after index pregnancy, %^e^	37.5	100	0.088^d^	-	-
Weight, kg	71.4 ± 14.0	89.5 ± 13.9	**0.005**	0.716	**0.044**
BMI, kg/m^2^	26.2 ± 4.5	32.9 ± 4.5	**0.001**	-	-
Weight change after index pregnancy, kg	−1.7 ± 7.8	5.0 ± 6.9	**0.048**	0.296	**0.044**
Waist circumference, cm	87.3 ± 9.4	98.8 ± 8.8	**0.004**	0.740	0.150
WHR	0.84 ± 0.07	0.85 ± 0.06	0.772	0.977	0.710
Waist-to-height ratio	0.53 ± 0.06	0.60 ± 0.05	**0.002**	0.402	0.317
FM, kg	24.1 ± 9.3	36.8 ± 10.2	**0.005**	0.540	0.539
FM, %	32.6 ± 6.9	40.5 ± 6.0	**0.009**	0.360	0.422
FFM, kg	47.4 ± 5.8	52.7 ± 6.0	**0.028**	0.311	0.505
FFM, %	67.4 ± 6.9	59.5 ± 6.0	**0.009**	0.620	**0.025**
Serum cholesterol, mmol/l	4.7 ± 0.8	4.5 ± 0.7	0.527	0.404	0.264
Serum HDL, mmol/l	1.5 ± 0.3	1.3 ± 0.2	0.107	0.759	0.950
Serum LDL, mmol/l	2.9 ± 0.9	2.9 ± 0.8	0.905	0.593	0.509
P-glucose change after index pregnancy, mmol/l	0.5 ± 1.3	1.1 ± 0.8	0.066	-	-
Serum insulin, mU/l	7.4 ± 4.6	15.4 ± 3.6	**0.001**	0.528	0.051
HbA1c					
mmol/mol	37.1 ± 3.4	41.5 ± 6.1	**0.037**	**0.027**	0.339
%	5.5 ± 0.3	5.9 ± 0.6	**0.037**	0.261	0.148
HOMA-IR	1.78 ± 1.13	4.35 ± 1.11	**0.001**	0.220	**0.029**
Serum hsCRP, mg/l^f^	1.8 ± 1.9	5.8 ± 6.6	0.067	0.089	**0.049**
Serum leptin, μg/l	17.6 ± 10.3	32.9 ± 15.9	**0.011**	0.142	0.157
Serum adiponectin, mg/l	11.6 ± 8.3	9.0 ± 3.8	0.441	0.970	0.974
Serum AFABP, μg/l	21.1 ± 7.7	34.1 ± 13.0	**0.006**	**0.021**	**0.010**

Values are mean ± SD. ^a^ Independent samples Mann-Whitney U test. ^b^ Adjusted for BMI by Mantel’s test ^c^ Adjusted for BMI and ethnicity by Mantel’s test ^d^ Fisher’s exact test. ^e^ Number of pregnancies after index pregnancy; 8 (NGT), 3 (IGM). ^f^ Two women with IGM had a CRP concentration > 10 mg/l (both 16 mg/l).

### Adipose tissue characteristics at follow-up

**Figure 1.**
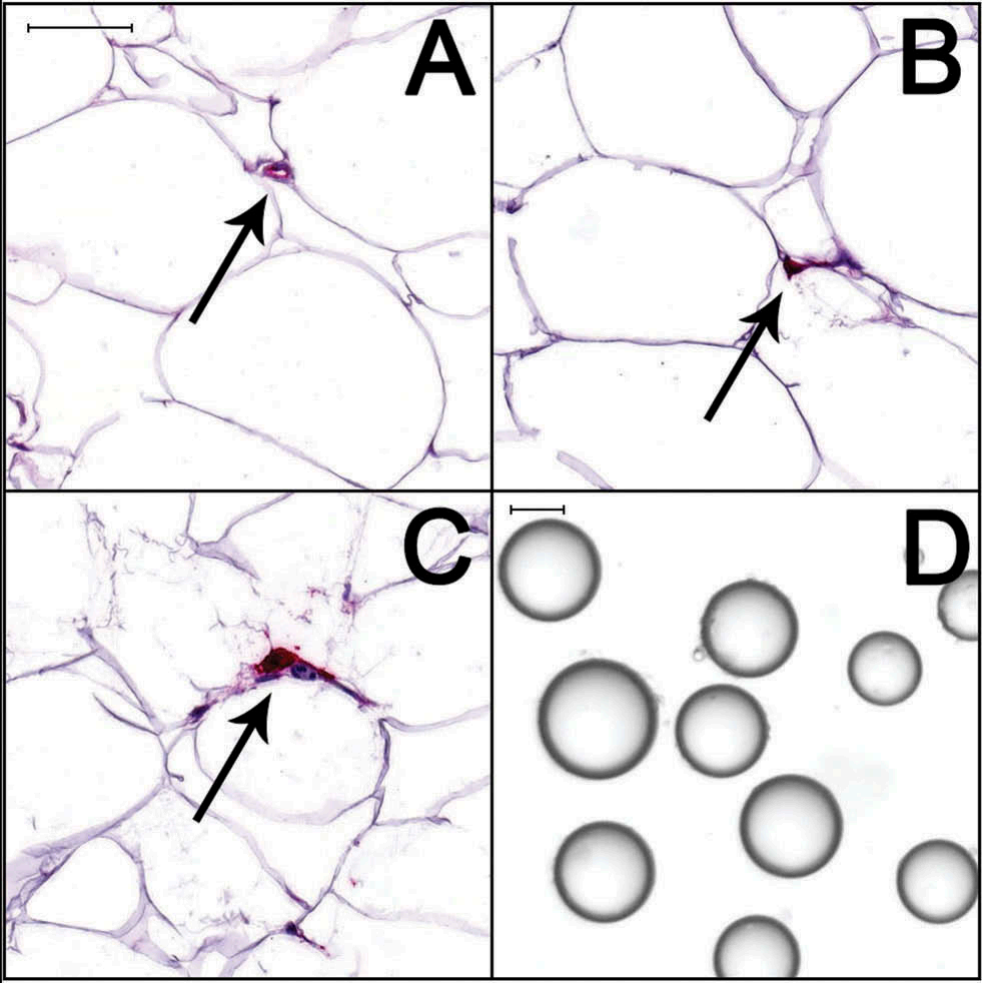
Adipose tissue sections (a–c) and isolated adipocytes (d) from a woman with NGT. The sections were stained with antibodies against von Willebrand factor to detect vessels (a), CD68 to detect macrophages (b), and mast cell tryptase to detect mast cells (c). Warp red was used as chromogen, and slides were counterstained with hematoxylin. Arrows indicate positive immunoreactions. Scale bars, 50 μm.

Adipose tissue characteristics of the women are shown in Table 3. Adipocytes were larger in women in the IGM group than in the NGT group. The number of adipocytes did not differ between the groups. Women with IGM had fewer small adipocytes and more large and very large adipocytes than women with NGT. The mean diameter of reference microspheres was 97.79 ± 0.12 μm (n = 14). In each cell population, 563−1180 adipocytes (mean 990 ± 135) were analyzed. Adipose tissue of women in the IGM group had a higher density of mast cells and greater release of tumor necrosis factor alpha (TNF-α) *in vitro* than adipose tissue of women in the NGT group. After adjustment for BMI, the adipose tissue release of TNF-α and vessel density were higher in the IGM group (Table 3). After adjustment for both BMI and ethnicity, there were no remaining differences between the groups (Table 3). The adipose tissue section area analyzed with immunohistochemistry was 70.4 ± 29.9 mm^2^ (NGT) and 83.3 ± 25.9 mm^2^ (IGM). Representative images of isolated cells and tissue sections are shown in Figure 1.

**Table 3.** Adipose tissue characteristics of women at follow-up 6 years after GDM diagnosis.

	GDM at index pregnancy (n = 39)				
Characteristic	NGT at follow-up (n = 31)	IGM at follow-up (n = 8)	*P* ^a^	*P adj ^b^*	*P adj ^c^*
**Cellularity**					
Adipocyte diameter, µm	82.3 ± 9.9	91.0 ± 15.5	0.123	0.686	0.090
Adipocyte volume, pl	440.4 ± 128.8	612.2 ± 229.8	**0.044**	0.547	0.056
Total adipocyte number, 10^10^	6.70 ± 3.57	7.53 ± 2.64	0.328	0.584	0.203
< 50 µm, %	25.0 ± 11.7	24.8 ± 11.2	0.986	0.978	0.221
50–100 μm, %	36.9 ± 14.3	21.5 ± 10.0	**0.007**	0.344	0.080
100–150 μm, %	37.5 ± 12.9	51.5 ± 17.4	**0.033**	0.621	0.174
> 150 μm, %	0.5 ± 1.4	2.2 ± 2.9	**0.015**	0.622	0.136
**Density**					
Macrophages, no./10^3^ adipocytes^d^	0.29 ± 0.69	0.63 ± 0.77	0.196	0.667	0.446
Mast cells, no./10^3^ adipocytes^d^	4.02 ± 3.03	7.82 ± 3.88	**0.012**	0.120	0.171
Vessels, µm^2^/adipocyte	53.8 ± 58.0	73.8 ± 63.8	0.382	**0.025**	0.051
**Release**					
Glycerol, nmol/10^4^ cells	7.7 ± 4.4	11.9 ± 6.4	0.130	0.539	0.153
IL-6 24h, ng/l/g^d^	412.4 ± 278.1	434.6 ± 230.1	0.928	0.409	0.255
TNF-α 24h, ng/l/g^d^	2.2 ± 1.3	3.4 ± 1.1	**0.033**	**0.010**	0.053
Adiponectin 24h, μg/l/g	158.6 ± 50.4	159.8 ± 43.0	0.871	0.130	0.352

Values are mean ± SD. ^a^ Independent samples Mann-Whitney U test. ^b^ Adjusted for BMI by Mantel’s test ^c^ Adjusted for BMI and ethnicity by Mantel’s test ^d^ Two women with IGM had a CRP concentration > 10 mg/l (both 16 mg/l).

### Multivariable regression

Stepwise multivariable logistic regression was done with IGM as the dichotomous dependent variable. All adipose tissue characteristics in Table 3, and the adipose tissue–related variables in Table 2 (BMI, weight, weight change after index pregnancy, waist circumference, waist-hip ratio (WHR), waist-to-height ratio, fat mass in absolute and relative terms, and serum levels of hsCRP, leptin, adiponectin, and AFABP), were introduced as independent variables. In the final analysis, a complete data set was available for 39 women. Follow-up BMI was associated with increased risk of belonging to the IGM group (BMI: β = 0.294; SE = 0.107; *P *= 0.006; OR = 1.342. Constant: β = –9.998; SE = 3.305; *P *= 0.002; OR = 0.000). Including the index pregnancy BMI in the analysis did not change the outcome.

Stepwise multivariable linear regression was done with HOMA-IR as the dependent variable. Adipose tissue–related variables that correlated with HOMA-IR were used as independent variables (Table 4). A complete data set was available for 38 women (data on HOMA-IR was missing in one woman). The best regression model included waist-to-height ratio and adipocyte volume and explained 65.6% of the variance in HOMA-IR (*P *< 0.001). The variable most strongly associated (highest correlation coefficient) with insulin sensitivity was waist-to-height ratio.

**Table 4.** Factors associated with insulin sensitivity in women at follow-up 6 years after GDM diagnosis.

Model	Variables	*β*	R^2^
I	Waist-to-height ratio	0.749**	0.549**
II	Waist-to-height ratio	0.589**	0.656**
	Adipocyte volume, pl	0.374*	

*P < 0.01, **P < 0.001; n = 38 Multivariable linear regression (stepwise) with HOMA-IR as the dependent variable. Independent variables: weight, BMI, weight change since index pregnancy, waist circumference, WHR, waist-to-height ratio, body fat mass (absolute value and in relation to body weight), serum levels of hsCRP, leptin, adiponectin, and AFABP, adipocyte volume, total number of adipocytes, numbers of adipocytes 50–100 μm, 100–150 μm, and > 150 μm in relation to total number, and glycerol release *in vitro*. The inclusion criterion of the model was an F probability of 0.05. The exclusion criterion was an F probability of 0.1. *β, β* standardized coefficient; R^2^, adjusted determination coefficient.

The proportions of small and large adipocytes correlated inversely with mast cell density, hsCRP and circulating levels of the adipokines leptin, adiponectin, and AFABP. (Table 5).

**Table 5.** Spearman correlations for adipocyte proportions (% of total adipocyte number) and inflammatory/metabolic markers in women at follow-up 6 years after GDM diagnosis.

	Very small	Small	Large	Very large
Serum leptin, μg/l	0.293	–0.509***	0.250	0.364*
Serum adiponectin, mg/l	–0.302	0.388*	–0.162	–0.451**
Serum AFABP, μg/l	0.156	−0.352*	0.225	0.336*
Serum hsCRP, mg/l	0.163	–0.528***	0.328*	0.464**
Mast cell density, no./10^3^ adipocytes	–0.062	–0.489**	0.387*	0.153
Macrophage density, no./10^3^ adipocytes	0.115	–0.232	0.098	0.162

*P < 0.05, **P < 0.01, ***P < 0.001; n = 39. Adipocytes classified as very small (< 50 μm diameter), small (50–100 μm), large (100–150 μm), and very large (> 150 μm).

## Discussion

In this follow-up study of women 6 years after GDM, we identified three factors – all related to adipose tissue and body composition – that independently of BMI and ethnicity may increase the risk of progression to T2DM after GDM. These factors were increased serum AFABP, weight gain after index pregnancy, and a low proportion of FFM. As expected, high BMI, abdominal fat distribution, and enlarged adipocytes with corresponding signs of inflammation were also associated with development of manifest diabetes mellitus after GDM.

Serum AFABP was the single factor consistently higher in the IGM group than in the NGT group, with or without adjustment for BMI and BMI/ethnicity. AFABP, also known as fatty acid–binding protein 4, is an adipokine released preferentially from adipocytes, as well as from macrophages and endothelial cells.^15^ Fatty acid–binding proteins are a family of cytoplasmic proteins that bind long-chain fatty acids and other hydrophobic ligands.^16^ The regulation and role of circulating AFABP in metabolic disease have not yet been defined. This adipokine is closely associated with obesity, is a marker of the metabolic syndrome, and has been reported to predict T2DM independently of obesity, insulin resistance, and glycemic indexes in a selected sample of a population-based cohort.^15,17^ Further, AFABP and adiponectin, leptin, and TNF-α have been suggested as the most probable adipokine candidates in the pathophysiology of GDM.^18^ However, in a recent cross-sectional analysis, AFABP was suggested to be a surrogate parameter of fat mass rather than being involved in the pathogenesis of metabolic syndrome.^19^ Future studies should address the question of whether AFABP contributes to the pathogenesis of metabolic disease states, including T2DM after GDM.

Consistent with our findings, weight gain during postpartum follow-up after GDM has previously been shown to significantly increase the risk of T2DM,^8,9^ underscoring the importance of weight maintenance or weight loss in these high-risk women. Well-designed trials are needed to assess the effects of lifestyle interventions on preventing subsequent progression to T2DM among women with a history of GDM.^20^

Women with IGM at follow-up in the present study had a lower proportion of FFM than women who remained normoglycemic. Skeletal muscle is the largest component of adipose tissue–free body mass in humans, and accounts for the majority of whole-body insulin-stimulated glucose uptake.^21,22^ Still, findings of a beneficial influence specifically of lean mass on metabolic health in adults, as suggested in the present study, are rare.^23,24^

In the studied group of 39 women as a whole, the proportions of small and large adipocytes consistently correlated inversely with circulating adipokines, CRP, and mast cell density; the large adipocytes correlated positively with AFABP, leptin, CRP, and mast cell density and negatively with adiponectin – the opposite of what was observed for the small adipocytes. These results strongly support the view that large adipocytes are associated with several features of insulin resistance and inflammation, whereas small adipocytes are protective.^25^ In accordance with this view, adipocyte volume together with vessel density and TNF-α release in adipose tissue tended to be higher in the IGM group than in the NGT group after adjustment for BMI/ethnicity.^26^

The advantage of this study is the comprehensive analysis of adipose tissue morphology and function, together with determination of body composition and blood chemistry. The computerized technique used to measure adipocyte size enabled us to study the proportions of adipocytes of specific sizes. The study also had limitations. The sample size is relatively small, and the majority of the findings are based on cross-sectional data. An adipose tissue biopsy and thorough phenotyping of the women at the index pregnancy would have been valuable. Further, after adjustment for BMI and ethnicity, body weight differed in the NGT and IGM groups. Thus, BMI may not be an optimal index of body composition when subjects of various ethnicities are analyzed together. Importantly, fat mass, both absolute and relative, waist circumference, WHR, and waist-to-height ratio were similar in the groups after adjustment for BMI/ethnicity. The proportion of women treated with insulin during pregnancy tended to be higher in the IGM group. In the present analysis, we were unable to adjust for this tendency.

To conclude, women with a history of GDM are at particularly high risk of developing T2DM. Identification of factors differentiating the women at highest risk is crucial from predictive/preventive and possibly therapeutic perspectives. AFABP emerged as an interesting factor in this context. Serum AFABP was the single factor consistently higher in the IGM group than in the NGT group, both before and after adjustment for BMI and BMI/ethnicity. Future studies should address whether AFABP is involved in the pathogenesis of metabolic disease states, including T2DM after GDM. Other risk factors identified in the present study are established in the pathogenesis of T2DM and emphasize the importance of weight maintenance or weight loss after GDM, particularly in women with central fat accumulation.

## Subjects and methods

### Subjects

Participants were recruited from a larger cohort of women diagnosed with GDM at Sahlgrenska University Hospital, Gothenburg, Sweden, between 2005 and 2007.^27^ The diagnosis of GDM at the index pregnancy was principally based on the 1991 criteria of the European Association for the Study of Diabetes:^28^ fasting plasma glucose ≥ 7.0 mmol/l, OGTT 2-hour plasma glucose ≥ 10.0 mmol/l, and random nonfasting plasma glucose ≥ 12.2 mmol/l. GDM was diagnosed if any of these values were elevated. Glucose was analyzed in capillary blood with a HemoCue Glucose 201+ Analyzer (HemoCue, Ängelholm, Sweden), and blood glucose concentrations were converted to equivalent plasma glucose concentrations.^29^

Between 5 and 6 years after GDM diagnosis, the women were contacted by telephone. Those interested in participating in a follow-up study received further information and an invitation by mail. Women who were pregnant at the time of follow-up were excluded. Participants who consented to an optional subcutaneous abdominal adipose tissue biopsy were eligible for the present study. Exclusion criteria were diagnosed type 1 and type 2 diabetes, other endocrine diseases that affect adipose tissue, malignancies, use of neuroleptic drugs, and gastric bypass surgery after the index pregnancy. In total, 39 women were included in the study.

The study was done at Sahlgrenska University Hospital, Gothenburg, Sweden. All participants received information about the study and gave oral and written consent. The study protocol was approved by the Regional Ethical Review Board in Gothenburg and was in accordance with the principles of the Declaration of Helsinki.

### Follow-up study procedure

Two study visits were scheduled. The first included an OGTT, anthropometric measurements, and blood sampling. The second visit included sampling of adipose tissue and determination of body composition. Participants were asked to fast overnight before study visits. Characteristics related to the index pregnancy with GDM diagnosis, and information on parity, previous GDM, ethnicity (European or non-European descent), and heredity were collected from medical records. In four women, OGTT data were missing, and the diagnosis was based on fasting or nonfasting random glucose; data on fasting glucose were missing in 3 women.

### Follow-up anthropometry and body composition

Waist and hip circumferences and height were measured by standard protocols to the nearest 1 cm and 0.5 cm, respectively. Body weight and volume, based on air-displacement plethysmography, were assessed with Bod Pod (software version 4.2.1 and 5.2.0; Cosmed, Rome, Italy), as described.^30^ Body density was calculated and converted to body composition based on the two-component model.^31^ Body volume was corrected for thoracic gas volume predicted by the Bod Pod software.^32^ Correction for the overestimation of body fat percentage in obese women by Siri’s formula^31^ was done as described.^33^

### Follow-up glucose tolerance and insulin sensitivity

Venous plasma glucose and serum insulin concentrations were determined in the fasted state and during a 75-g OGTT. Fasting and OGTT 2-h glucose concentrations were used to classify women as having NGT (fasting glucose < 6.1 mmol/l and 2-h glucose < 7.8 mmol/l), IFG (6.1≤ fasting glucose < 7.0 mmol/l and 2-h glucose < 7.8 mmol/l), IGT (fasting glucose < 7.0 mmol/l and 7.8 ≤ 2-h glucose < 11.1 mmol/l), or type 2 diabetes (fasting glucose ≥ 7.0 mmol/l or 2-h glucose ≥ 11.1 mmol/l), based on the 1999 criteria of the WHO.^34^ IGM was defined as the presence of IFG, IGT, combined IFG/IGT, or previously undiagnosed type 2 diabetes.

HOMA-IR, which correlates well with insulin sensitivity measured by the hyperinsulinemic euglycemic clamp technique (R = 0.88, *P *< 0.0001),^35^ was calculated as fasting insulin (mU/l) x fasting glucose (mmol/l)/22.5.^35^ HOMA-IR was not calculated in one woman with IGM, owing to hemolysis of her serum sample.

### Adipose tissue biopsy

Under local anesthesia, an abdominal subcutaneous adipose tissue biopsy was obtained by needle aspiration from the hypogastric region at a point one-third of the distance from the umbilicus to the superior anterior iliac spine. The biopsy was prepared as described below.

### *Adipokine release* in vitro

Adipokine release was studied in explants of adipose tissue incubated under sterile conditions as described.^36^ In brief, about 250 mg of adipose tissue was incubated in 10 ml of medium. After 4 h, the medium was separated from the tissue, centrifuged for 5 min at 450 g, and stored at – 80°C. Fresh medium (10 ml) was added to the tissue; 20 h later, the medium was removed, centrifuged, and stored as above. Adipokine concentrations were expressed per liter of medium and per gram of adipose tissue. Adipokine release over 24 h was calculated as the sum of the concentrations in the two samples. For logistic reasons, adipokine release *in vitro* was not determined in two women with NGT.

### Adipocyte size and number

Adipose tissue was digested with collagenase (Type A; Roche, Mannheim, Germany); an adipocyte suspension was prepared, and adipocyte size was determined by computerized image analysis (Leica QWin V3; Leica Microsystems, Wetzlar, Germany).^36–38^ Twelve random visual fields were photographed (DFC320, Leica Microsystems). Microspheres 98 μm in diameter (Dynal, Invitrogen, Oslo, Norway) served as reference. Adipocytes were classified as very small (< 50 μm diameter; d), small (50–100 μm), large (100–150 μm), and very large (> 150 μm). The formulas for the cross-sectional area (πd^2^/4) and the volume (πd^3^/6) of a sphere were used to calculate the size and weight (density 0.9 g/ml) of individual adipocytes. In calculations of immune cell and vessel density, and adipocyte glycerol release, the sample adipocyte size/weight distribution was used. Mean adipocyte volume was determined as the average of all adipocyte volumes. Body adipocyte number was estimated by using body fat volume and the adipocyte volume distribution.

### Lipolysis

Isolated adipocytes were incubated for 2 h, and glycerol released into the medium was determined as an index of basal lipolysis as described.^39^ The amount of triglycerides in the adipocyte suspension was measured after extraction and evaporation of solvents.^40^ Incubations were done in duplicate. Lipolytic activity was expressed as nmol per 10^4^ adipocytes. For logistic reasons, lipolytic activity was not determined in one woman with NGT.

### Immunohistochemistry

Part of each biopsy was fixed in phosphate-buffered formalin, processed (Leica TP 1020, Leica Microsystems), embedded in paraffin, and cut into 5-μm-thick sections. The sections were subjected to high-temperature antigen retrieval for 10 min in EDTA buffer, pH 8, followed by blocking and incubation with antibodies against macrophages (Monoclonal mouse anti-human CD68, clone PG-M1, M0876, Dako, Glostrup, Denmark), mast cells (monoclonal mouse anti-human mast cell tryptase, clone AA1, M7052, Dako), and von Willebrand factor (polyclonal rabbit anti-human von Willebrand Factor, A0082, Dako) to detect vessels. For visualization of immunoreactions, MACH3 mouse AP-polymer (M3M532) was used for macrophages and mast cells and MACH3 Rabbit AP-Polymer (M3R533) for vessels (Biocare Medical, Concord, CA). The chromogen was Warp Red (WR806, Biocare Medical). Slides were counterstained with hematoxylin, mounted, scanned with a Mirax Digital Desk Scanner (Zeiss, Göttingen, Germany), and analyzed with Tissue Studio 3.6.1 (Definiens, Munich, Germany). Macrophage and mast cell densities were expressed as the number of immunoreactive cells per 10^3^ adipocytes. Vessel density was expressed as positive signal area per adipocyte.

### Biochemical assays

HbA1c was analyzed with a point-of-care analyzer (Afinion AS100; Axis-Shield, Oslo, Norway) during the first study visit. All other blood and medium samples were analyzed at the Clinical Chemistry Laboratory, Sahlgrenska University Hospital, which is accredited in accordance with the International Standard ISO 15189:2007. ELISA was used to measure adiponectin (human adiponectin ELISA kit, EZHADP-61K, Millipore, Billerica, MA; interassay coefficient of variation (CV) 7.0% at 10.5 mg/l), TNF-α (human TNF-alpha Quantikine HS ELISA, HSTA00D, R&D Systems, Minneapolis, MN; interassay CV 6.0% at 2 ng/l), AFABP (human adipocyte FABP ELISA kit, RD191036200R, BioVendor Research and Diagnostic Products, Bratislava, Slovakia; inter-assay CV 10.0% at 16 μg/l) and leptin (human leptin Quantikine, DLP00, R&D Systems; interassay CV 8.0% at 9 μg/l). Glycerol was analyzed with a Randox radiometric glycerol kit (GY105; Crumlin, UK; interassay CV 4.0% at 0.1 μmol/l) and a Konelab 30 autoanalyzer (Thermo Clinical Labsystems, Vantaa, Finland). All other assays were routinely done with a Cobas Modular system (Roche Diagnostics, Mannheim, Germany).

### Statistical analysis

Values are mean ± SD (continuous variables) or percentage (categorical variables). The independent-samples Mann-Whitney U test, χ^2^ test, and Fisher’s exact test were used to compare NGT and IGM groups. Mantel’s test was used to eliminate the influence of BMI or both BMI and ethnicity on the studied variables.^41^ BMI was divided into four groups (22–26, 26–28, 28–32, 32–40 kg/m^2^) and ethnicity was simultaneously divided in two groups (European/non-European descent). Within each group, the correlations between the zero-one variable NGT/IGM and the other variables were studied. The results of each BMI and ethnicity group were pooled to result in a *P* value. Multivariable logistic regression was used to determine the probability of belonging to the IGM group. Multivariable linear regression was used to identify determinants of HOMA-IR. Adipose tissue–related variables that correlated with HOMA-IR by Spearman’s rank-correlation test were selected as independent variables in linear regression analysis. Spearman’s rank-correlation test was also used to evaluate associations between adipocyte proportions and immune cell densities, serum hsCRP, and concentrations of adipokines. *P *< 0.05 was considered significant. All statistical analyses were done in SPSS (v. 21 for MacOS X, SPSS, Chicago, IL) except Mantel’s test (an extension of Fisher’s permutation test and Pitman’s test), which was done with a non-commercial program made by professor Anders Odén (Department of Mathematical Sciences, Chalmers University of Technology). The *P* value was approximated with the Edgeworth expansion. Power properties of Fisher’s permutation test were as described.^42^

## Abbreviations

AFABP,: adipocyte fatty acid-binding protein
BMI,: body mass index
FFM,: fat free mass
FM,: fat mass
GDM,: gestational diabetes mellitus
HbA1c,: glycated hemoglobin
hsCRP,: high-sensitivity C-reactive protein
HOMA-IR,: homeostasis model assessment of insulin resistance
IFG,: impaired fasting glucose
IGM,: impaired glucose metabolism
IGT,: impaired glucose tolerance
NGT,: normal glucose tolerance
OGTT,: oral glucose tolerance test
TNF-α,: tumor necrosis factor alpha
T2DM,: type 2 diabetes mellitus
WHR,: waist-to-hip ratio
